# Rapid Elimination of *Aedes aegypti* and *Culex quinquefasciatus* Mosquitoes from Puerco Island, Palawan, Philippines with Odor-Baited Traps

**DOI:** 10.3390/insects14090730

**Published:** 2023-08-28

**Authors:** Bart G. J. Knols, Arnel Posada, Mark J. Sison, Johan M. H. Knols, Nila F. A. Patty, Akib Jahir

**Affiliations:** 1 K&S Consulting, Kalkestraat 20, 6669 CP Dodewaard, The Netherlands; 2 Ecoresort Development Corporation, Purok Bagong Silang, Poblacion 1, Roxas 5308, Palawan, Philippines; 3 Biogents AG, Weißenburgerstraße 22, 93055 Regensburg, Germany; 4 Soneva Fushi, 4th Floor Jazeera Building, Boduthakurufaanu Magu, Male 20077, Maldives

**Keywords:** mosquito, *Aedes*, *Culex*, arbovirus, elimination, island, trapping, Philippines

## Abstract

**Simple Summary:**

Traps baited with attractants that mimic the smell of a human being have been shown to be a suitable, pesticide-free, alternative for mosquito control. Here we report the outcome of a trial on an island in the Philippines, where a density of 10 traps per hectare (72 in total) resulted in a surprisingly fast decline in populations of two mosquito species and their subsequent elimination within five months. This is the second time that odor-baited traps have succeeded in eliminating mosquitoes from a small tropical island. High-density trapping is an environmentally-friendly, pesticide-free, cost-effective and mosquito-specific approach to control or eliminate mosquito nuisance biting and disease risk for people living in (small) islands in the tropics.

**Abstract:**

Globalization and climate change are key drivers for arboviral and parasitic infectious diseases to expand geographically, posing a growing threat to human health and biodiversity. New non-pesticidal approaches are urgently needed because of increasing insecticide resistance and the negative human and environmental health impacts of synthetic pyrethroids used for fogging. Here, we report the complete and rapid removal of two mosquito species (*Aedes aegypti* L. and *Culex quinquefasciatus* Say), both arboviral disease vectors, with odor-baited mosquito traps (at a density of 10 traps/hectare) from a 7.2-hectare island in the Philippines in just 5 months. This rapid elimination of mosquitoes from an island is remarkable and provides further proof that high-density mosquito trapping can play a significant role in mosquito- and vector-borne disease elimination in small islands around the world.

## 1. Introduction

Arthropod-borne viruses (arboviruses), notably dengue fever, are rapidly expanding mosquito-transmitted viral diseases that exert a huge economic and health burden in major parts of the tropics and sub-tropics [1]. For dengue fever alone, the incidence has increased 30-fold since the 1970s, and the disease is now estimated to affect more than 390 million people annually [1,2]. This increase in prevalence is due to substantial growth in urbanization, international travel and trade, climate change, geographical expansion of its major mosquito vectors, and failure of current (mostly insecticide-based) control methods [3]. Recently, alarming levels of insecticide resistance were reported from southeast Asia, and concerns arose that underlying mutations may spread rapidly within the region and beyond over the next few years. This would result in the loss of the most common approach to control mosquitoes in this part of the world: insecticide fogging [4]. As a consequence, the high infection rate of the disease and the resulting toll on development and economic progress through lost productivity and costs of mosquito control activities are likely to increase in the foreseeable future [3,4,5,6].

In recent years, the sterile or incompatible insect technique (SIT, IIT), the combination of both methods, based on radiation-based sterilization or genetic modification [7], and the use of *Wolbachia* bacteria to induce sterility or refractoriness to arboviruses in mosquito populations [8,9] have been piloted in various parts of the world. Reductions in target mosquito populations have been reported to exceed 90% [8,9,10,11]. Disadvantages of these approaches are the cost and the need for a mass production facility for mosquitoes, the need for sexing, and the complexity of transporting and releasing millions of male mosquitoes over large areas. These technical challenges and the cost-effectiveness may be manageable in high-density human population settings [12] but are difficult to justify in (remote) island settings, where this approach may not be economically feasible. Autodissemination traps, whereby the egg-laying female is contaminated with a larvicide (e.g., pyriproxyfen) that she carries to other breeding sites [13,14], have been developed but their effectiveness in terms of controlling disease transmission remains unknown. In contrast, biological control trials in Vietnam that deployed the predacious copepod *Mesocyclops* in water storage containers successfully reduced *Aedes* populations to near extinction and halted dengue transmission [15,16]. Although odor-baited traps for host-seeking mosquitoes were successfully deployed to reduce malaria on an island in Lake Victoria, Kenya [17], such trials were only recently undertaken against the Asian tiger mosquito (*Aedes albopictus*, Skuse) and southern house mosquito (*Culex quinquefasciatus* Say) in geographically isolated islands of the Maldives [18]. In these trials, suppression levels were comparable to those reported from genetic control trials but at a much lower cost. On Kunfunadhoo island (41.4 ha in size), peak suppression reached 93.0% (95% CI 91.7–94.4) for *Ae. albopictus* and 98.3% (95% CI 97.0–99.5) for *Cx. quinquefasciatus* at a total direct cost of USD 2056/ha/year when odor-baited BG-MosquitaireCO_2_ traps were used at a density of 6 traps/ha. On a smaller island (1.6 ha), Thahigandu Kolhu, *Ae. albopictus* was eliminated when the trap density reached 18.8 traps/ha [18]. The cost-effectiveness and insecticide-free nature of this approach sparked interest from other islands; here, we report the first trial on an island in the Philippines, against two mosquito species, the yellow fever mosquito (*Ae. aegypti*) and southern house mosquito (*Cx. quinquefasciatus*).

## 2. Materials and Methods

### 2.1. Study Island

**Figure 1:**
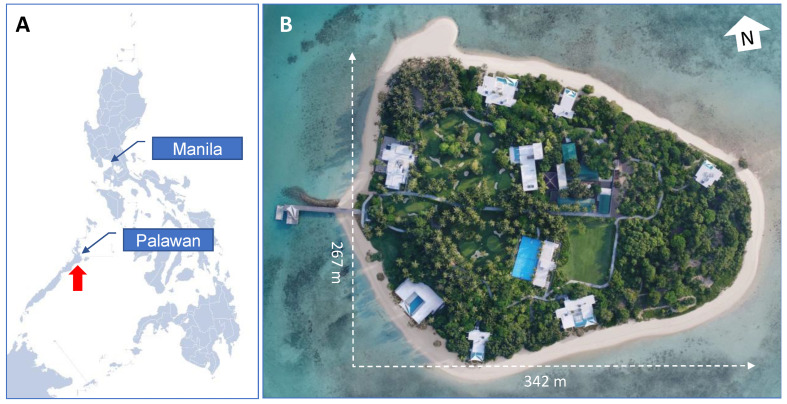
Location of Puerco Island, Palawan, Philippines. (**A**) Map of the Philippines, showing the location of its capital Manila and Palawan Island. The location of Puerco Island is shown by the red arrow. (**B**) Google Earth satellite image of Puerco Island (Imagery date 6 August 2022). The geographical center of the island is located at 10°19′07.07″ N, 119°28′53.14″ E.

The trial was conducted on Puerco Island, a 7.2-ha privately owned island (www.banwaprivateisland.com; accessed on 26 August 2022) situated 5.5 km off the north-east coast of Roxas, Palawan, in the Sulu Sea (Figure 1).

Only two mosquito species, *Ae. aegypti* and *Cx. quinquefasciatus* were found to be present on the island, in roughly a density of 5:1, respectively [19]. The island harbors a lush tropical vegetation consisting of coconut trees (*Cocos nucifera*), elephant ear (*Colocasia esculenta*), frangipani (*Plumeria* spp.), and various ornamental plants. The eastern part of the island consists of a bird sanctuary, mainly to protect a breeding area of the rare Tabon scrubfowl (*Megapodius cumingii cumingii*) [20]. Its climate is tropical rainforest, with an average annual temperature of 29 °C and a relative humidity >80%. Precipitation occurs throughout the year, with less rainfall in the months of December–April, and resulting lower mosquito numbers during that period. A weather station on the island was used to record rainfall.

The island can host a maximum of 48 guests and has ca. 100 staff, of which the majority commute to/from the mainland on a daily basis.

### 2.2. Mosquito Traps and Odor Baits

**Figure 2:**
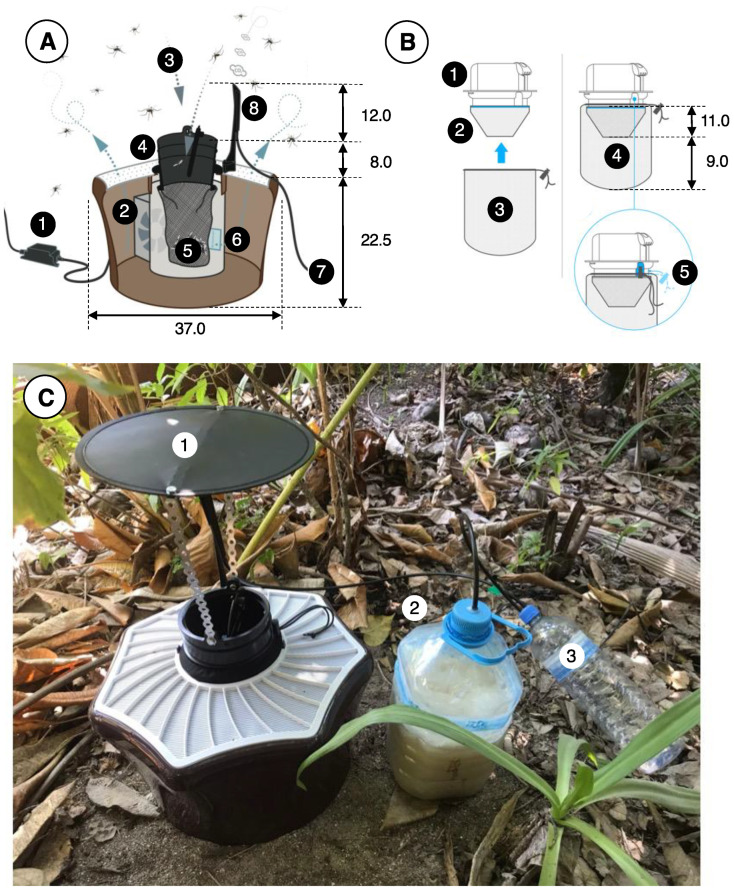
The BG-MosquitaireCO_2_ trap and its operation. (**A**) An adapter (1) converts power from the mains to 12 V DC (0.3 A, 3.6 W) that provides power to a fan (2) located inside the trap. Suction (ca. 3 m/s) by the fan creates an inward airflow (3) through the black trap inlet (4) and the catch bag (5, see (**B**)), and odor-laden air (with lactic acid emanating from a sachet) (6) leaves the trap via its perforated white top. Carbon dioxide is provided though 6 mm plastic tubing (7) and released from a nozzle at the top of the trap (8). (**B**) The inlet (1) of the trap has two netting bags attached to it, a so-called funnel bag (2), which prevents mosquitoes from flying upward, and the catch bag (3). Both bags (4) are fitted on the inlet (5) using an elastic string. (**C**) A trap in the field, fitted with a roof (1) to prevent rain from entering the trap. The 5 L water bottle (2) with water, sugar, and yeast to produce CO_2_ is located next to the trap. A 1.5 L overflow bottle (3) prevents liquid/foam from this mixture to enter the tubes and nozzle that might otherwise get clogged and disrupt the flow of CO_2_. Tubes are fitted to the polyethylene bottle tops using hot glue. All dimensions are in cm.

BG-MosquitaireCO_2_ traps (Figure 2) (Biogents AG, Regensburg, Germany) lure mosquitoes from within a range of ca. 20 m downwind of the trap [21]. Mosquitoes fly upwind in the trap’s odor plume and, once they arrive in its vicinity, become visually attracted by the contrasting colors of the trap components, notably the white cover top and central black inlet of the trap. When close to the trap, mosquitoes get sucked into the central black inlet through air suction produced by a 12 V DC fan located inside the trap and are then caught in a netting catch bag where they die of dehydration.

In order to attract host-seeking *Ae. aegypti* and *Cx. quinquefasciatus* females, as well as mate-seeking male *Ae. aegypti*, BG-MosquitaireCO_2_ traps were baited with sugar-fermenting yeast as an organic source of carbon dioxide (CO_2_) [18,22,23]. For every trap, a 5 L plastic water bottle was filled with 3 L of water, to which 700 g of white (granulated) sugar and 20 g of baker’s yeast, was added. Carbon dioxide measurements revealed that this mixture produces a concentration of ca. 70 mL/min after 12 h and 11–13 mL/min of after 72 h, when it would be replaced [18]. A second attractant (BG-Mozzibait^®^, Biogents AG, Regensburg, Germany) consisted of a sachet filled with beads impregnated with lactic acid [24,25]. These sachets were placed inside the trap and were replaced every fortnight.

**Figure 3:**
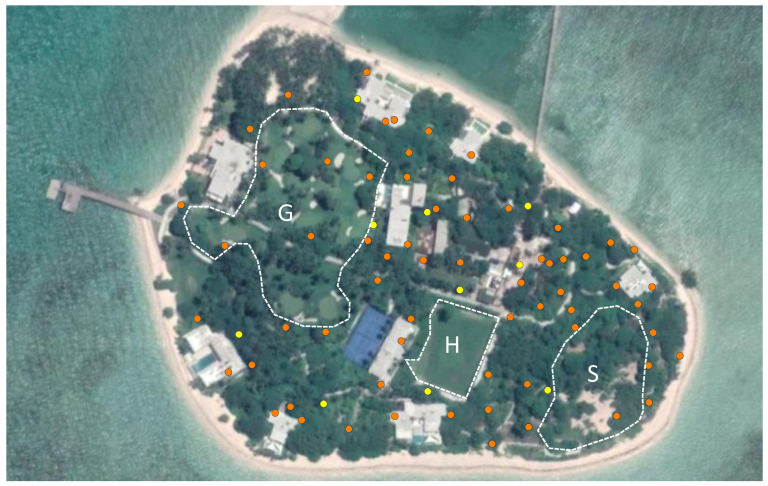
Locations of the 75 BG-MosquitaireCO_2_ traps on Puerco Island (orange), of which 10 traps served as monitoring traps (yellow). Areas with low-density traps are the golf course (G), the helipad (H), and the bird sanctuary (S).

A total of 72 traps were deployed between 5 and 9 July 2022, with an additional 3 (spare) traps on 19 November 2022, according to manufacturer recommendations (Figure 3). Traps were never placed in open sunlight and generally placed in shaded areas under vegetation cover. All traps were visited every third day in order to replace the bottles with the sugar/yeast solution. Across the island, 10 traps were selected as monitoring traps, and their catches were removed every third day, before being placed in a −5 °C freezer for 45–60 min to kill any remaining live mosquitoes, after which catches were counted by species and recorded in an online database. From the middle of October onward, at the time when sachets were being replaced in all traps (every fortnight), catches from all 75 traps were retrieved and counted.

Although limited in scope, some larval source management (LSM) activities were undertaken. No other mosquito control measures were practiced for the entire duration of the trial. Obvious (potential) breeding sites for mosquitoes were inspected and removed where possible. Occasionally, tablets containing Spinosad (Natular^TM^, Leads Environmental Health Products Corp., Santa Rosa, Philippines) [26,27] were used in water tanks or other water collections that could not be removed. Examples of (potential) breeding sites and the management thereof are shown in Supplementary Material Figure S1.

## 3. Results

**Figure 4:**
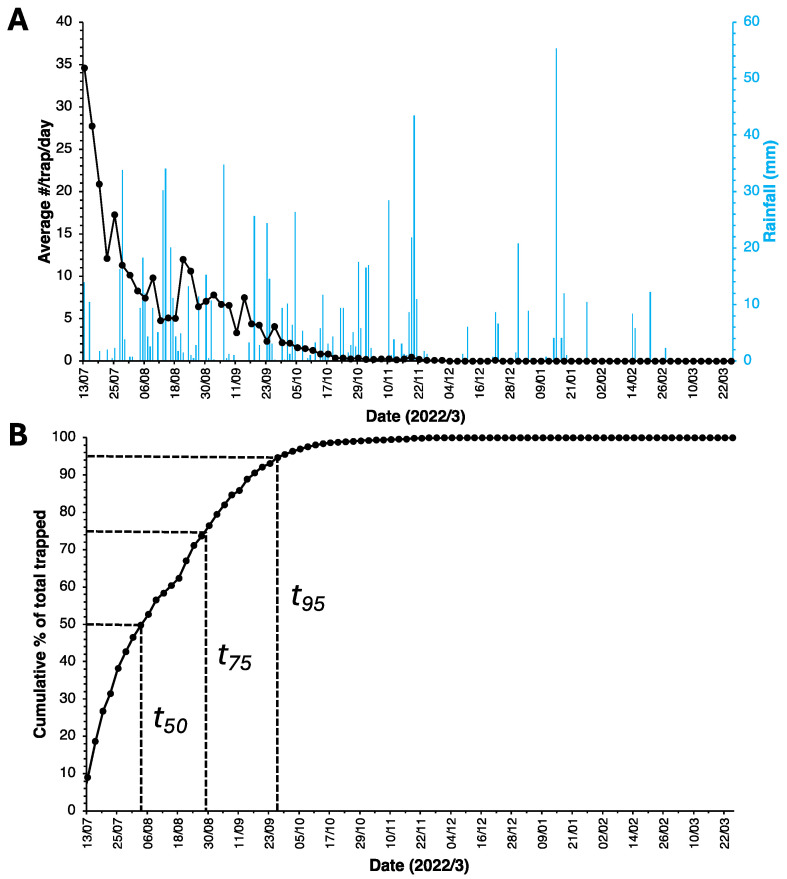
(**A**) Average number of mosquitoes (data pooled for both species) caught per day per (monitoring) trap between 13 July 2022 and 25 March 2023 on Puerco Island, Palawan, Philippines. Blue bars show rainfall (in mm) per day. (**B**) Cumulative percentage of mosquitoes removed from Puerco Island over the entire 6-month period (n = 6920), showing the time needed to trap 50% (t_50_), 75% (t_75_) or 95% (t_95_) of all mosquitoes collected for both species.

Following installation of the traps, an immediate and sharp drop in mosquito numbers was observed (Figure 4A). Initially, catches for both species were pooled and not counted separately. Catches of non-target insects were rare. Between 13 July 2022 and 9 January 2023, a total of 6920 mosquitoes were collected in the 10 monitoring traps.

In spite of significant rainfall in the months following trap installation, catch numbers dwindled and reached an average of just below 1 (97.4% reduction) after just 3 months of trapping. All monitoring traps were empty for the first time on 4 December, and, apart from three *Culex* mosquitoes collected on 22 December, all monitoring trap catches remained zero until the time this article was submitted for publication (end of July 2023). Figure 4B shows that half of all the mosquitoes collected (*t*_50_) were trapped in the first 23 days. For *t*_75_ and *t*_95_, these figures were 49 and 77 days, respectively. All trap data for the (monitoring) traps are shown in Supplementary Data S1.

**Figure 5:**
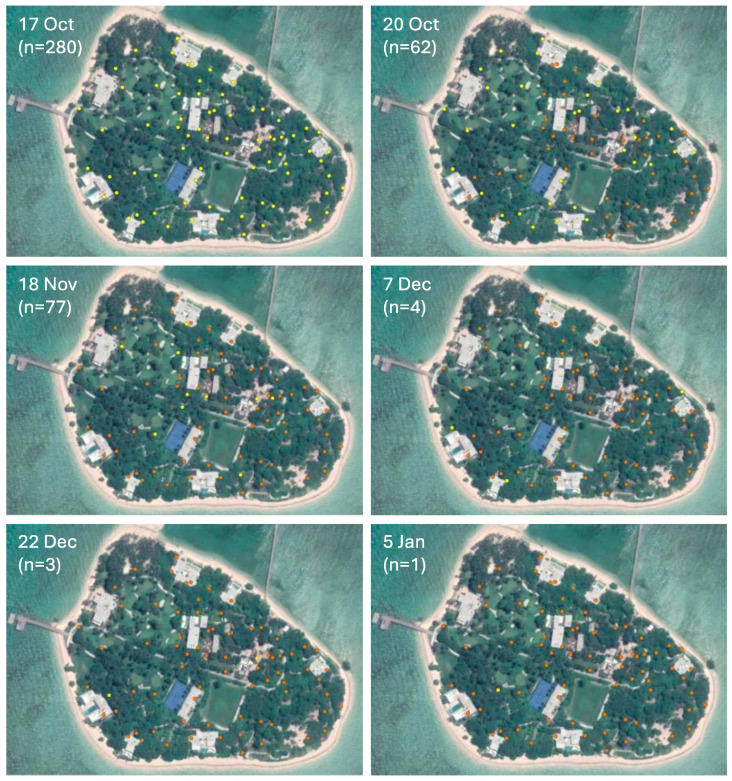
Traps positive (yellow symbols) or negative (orange) for mosquitoes between October 2022 and January 2023. Dates of collection are shown, as well as the total catch (in brackets).

From the middle of October onward, all 75 traps were emptied, and mosquitoes counted to closely monitor where their presence remained (Figure 5). It can be seen from these data that mosquitoes first started disappearing from the bird sanctuary area in the eastern part of the island. The last two mosquitoes (both *Culex*) from non-monitoring traps were caught sometime between 5 January and 23 February 2023. No mosquitoes were caught afterward until the time this article was submitted for publication (end of July 2023).

## 4. Discussion

Here, we report the rapid and complete removal of two mosquito species from an island in the Philippines with high-density (10 traps/ha) mosquito trapping. This is, to our knowledge, the first successful elimination of these disease vectors using this approach in southeast Asia. The speed at which elimination occurred is remarkable and unique in itself, and it holds promise for many small islands around the world that face both nuisance biting and disease risk posed by these vectors. The only other example of mass trapping for successful elimination is that of the Asian tiger mosquito, *Aedes albopictus*, on Thahigandu Kolhu, a small island in the Maldives, which has been free of this mosquito for 17 months (since February 2022) [18].

Although elimination is apparent based on trap catches, egg survival through desiccation resistance is a well-known phenomenon for *Ae. aegypti* (not for *Cx. quinquefasciatus*), with periods of egg survival depending on temperature and relative humidity, as well as the strain and geographical origin of mosquitoes reaching up to one year [28,29,30]. Thus, in principle, there could still be viable mosquito eggs on the island that may appear as adults later in time. What argues against this is the fact that there has been no drought period on the island, with rains continuing (Figure 4A), and any remaining eggs would, therefore, have been exposed to rain water and should have hatched.

As for previous studies [18,31], we acknowledge a shortcoming in our work to be the absence of a ‘control’. It does, however, seem very unlikely that both mosquito species would suddenly disappear from the island due to a factor other than the intervention (i.e., trapping). Moreover, although the uniqueness of each island questions the validity of a second island to serve as a control, we considered it inappropriate to have a second island without an intervention, thereby exposing inhabitants to nuisance biting and possibly mosquito-borne disease. Similarly, we could have opted for an experimental design whereby we significantly reduced the mosquito populations with trapping and then switched off the traps to let the populations recover. Following this, another period of intense trapping with the same outcome would add more certainty that it was the intervention that caused the observed effects. Again, this would be considered unethical and unacceptable to the island owners. Lastly, considering the importance that the World Health Organization assigns to an epidemiological endpoint when developing new vector control interventions [32], elimination should of course be convincing, i.e., zero mosquitoes equal zero risk of disease transmission. We conclude that, from a strictly scientific point of view, in the absence of a ‘control island’, we cannot claim that elimination was (exclusively) due to trapping even though the likelihood that trapping caused this is outcome remains high. The approach described here was implemented on 11 small Maldivian islands, on all of which the mosquito populations crashed, adding to the likelihood that the observed effect was due to high-density trapping [18].

Elimination has several strong benefits over control of mosquito populations. First, a time-limited operation with elimination as the outcome reduces the risk of evolution resulting in behavioral resistance, which long-term exposure of mosquitoes to traps and lures might induce, similarly to long-term exposure to insecticides [33]. Second, investment in elimination always yields a positive payback, i.e., in the long term, the cost savings will exceed the elimination costs. Similar trials in the Maldives had a direct cost of USD 2056 per hectare/year, which was 22.6% cheaper than fogging/misting [18]. On Puerco Island, the trap density was higher (10 vs. 6/ha) and the cost of attractants was higher, giving a direct cost of USD 2838 per hectare/year. If elimination takes 6 months, followed by 6 months post-elimination surveillance (using all traps), the cost would, thus, be USD 20,433. Third, time-limited operations have the benefit of strong support and enthusiasm from local communities and the operational/management teams, which may wane over time and result in complacency and a possible rebound of mosquito populations. Lastly, elimination brings the benefit that most traps can be removed and used elsewhere, perhaps apart from a few strategically placed monitoring traps that should be used to detect any possible reinvasion of mosquitoes.

Successful elimination brings two important considerations: the risk of reinvasion and impact on the ecosystem and natural enemies/predators. With regard to reinvasion, the biggest risk is posed by boat transport from the mainland to the island. It is very likely that some mosquitoes may have been transported to the island in the past, and that this will continue given the daily trips to/from the mainland. However, it is likely that the minimum founder population size will not be reached (estimated at 50–500 reproducing females with sufficient genetic heterogeneity) and, hence, will undergo genetic bottlenecking that results in the crash of populations after invasion [34]. Perhaps only when a large number of eggs are introduced (e.g., through the introduction of used car tires in which many females laid hundreds or thousands of eggs) could this be overcome and an invasion be successful. Considering potential negative consequences of removing mosquito species from ecosystems, there appears to be consensus that their role in nature is fairly limited [35]. For instance, no studies indicate that any predator exclusively depends on larvae of *Aedes aegypti* or *Ae. albopictus* for their survival; as adults, foraging theory predicts that these mosquitoes are of low profitability to predators [36]. Moreover, the impact of chemical spraying (fogging and misting), which is still the predominant practice of controlling mosquitoes throughout much of Asia, will be much greater and affect insect biodiversity to a much larger extent than trapping of one or a few mosquito species. Our studies in the Maldives [18] showed that, after 1.5 years without spraying of insecticides, a broad array of butterflies, dragonflies, bumblebees, and carpenter bees increased in numbers and contributed to restoring balance in these fragile ecosystems. Moreover, ending the spraying of synthetic pyrethroids, the dominant insecticides used for fogging against mosquitoes, will benefit corals and marine life for which these chemicals are extremely toxic [37]. High-density mosquito trapping will, therefore, benefit both marine and terrestrial biodiversity, particularly insect diversity, which is globally under threat [38].

Compared to contemporary genetic control strategies, trapping has several explicit advantages: (a) with traps, one targets both species of mosquito (*Aedes* and *Culex*) whereas genetic control trials target only one species; (b) trapping and LSM can be undertaken by personnel after only minimal training. The approach can, therefore, easily be expanded to other parts of the world where these mosquito species prevail. This is very different for genetic control trials that require highly skilled personnel; (c) trapping does not require the construction of mass-rearing facilities needed for genetic control trials, which are very expensive and again require specialized skills and expertise; (d) trapping is very much a ‘seeing is believing’ approach, and it quickly results in positive responses from the community when seeing catch bags filled with large numbers of trapped mosquitoes. Genetic control trials, in contrast, are based on the release of mosquitoes in the environment, which, certainly in the case of genetically engineered mosquitoes, has resulted in incidents of strong (public) opposition to such trials [39]; (e) trapping and LSM are significantly cheaper than genetic control trials. Nevertheless, although high-density trapping has all these advantages, operations over larger areas, including those which are not geographically and physically isolated, will easily become operationally complex and demanding in terms of transport, personnel, cost, etc. Larger, non-isolated areas can benefit from barrier placement of traps, which has been successfully trialed in Europe [40] and there is scope for combining trapping/LSM with genetic approaches, which we are currently exploring. For instance, considering that flooding with sterile or *Wolbachia*-carrying male mosquitoes normally requires a 10:1 release ratio (10 released males for every wild male), trapping/LSM can be used to lower populations before releases and serve as monitoring stations for the combined approach thereafter.

Given the problems with chemical control (e.g., fogging), notably insecticide resistance [4], there is growing interest in alternative approaches. High-density trapping, possibly in combination with genetic control approaches, may fulfill that need.

## 5. Conclusions

Odor-baited mosquito traps, used in high density (10 traps/ha), eliminated the yellow fever mosquito, *Aedes aegypti*, and the southern house mosquito *Culex quinquefasciatus*, from a 7.2-ha island in the Philippines over a period of 5 months. This approach offers a quick and, therefore, affordable opportunity for many small, inhabited islands around the world where these mosquito species pose a risk of arboviral disease transmission, as well as nuisance biting.

## Data Availability

Raw trapping data are provided in Supplementary Data S1.

## Supplementary Materials

The following supporting information can be downloaded at https://www.mdpi.com/article/10.3390/insects14090730/s1: Figure S1. Mosquito breeding sites and management practices; Data S1. Trap catch data.
