# Supplementary material for: Rapid Elimination of Aedes aegypti and Culex quinquefasciatus Mosquitoes from Puerco Island, Palawan, Philippines with Odor-Baited Traps
Source: Insects. 2023 Aug 28;14(9):730. doi: 10.3390/insects14090730 (PMC10531793; doi:10.3390/insects14090730)
Supplement: Supplementary file 1 [file insects-14-00730-s001.zip › Knols et al._Figure S1.pdf]

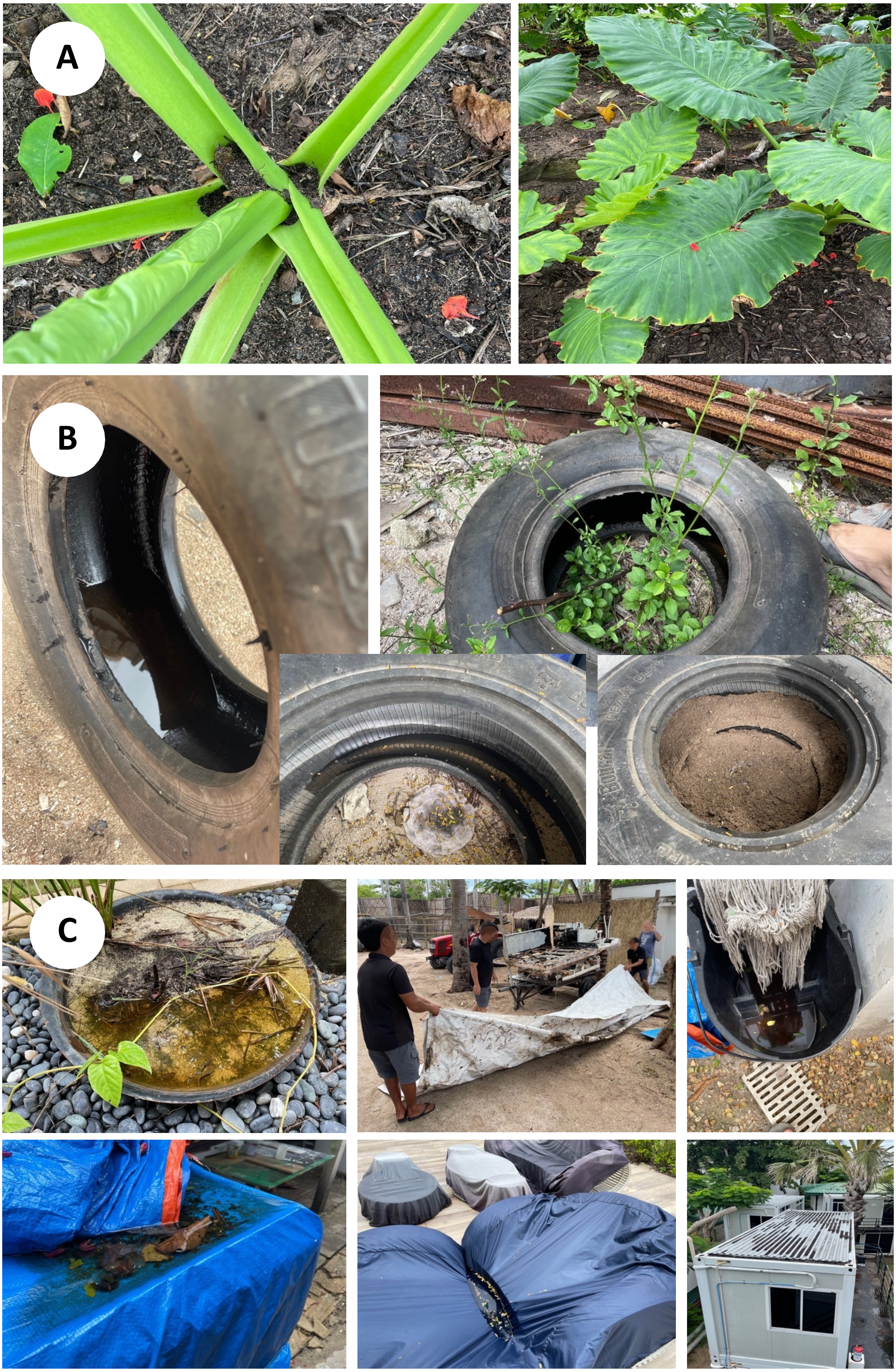

(Figure legend see next page)

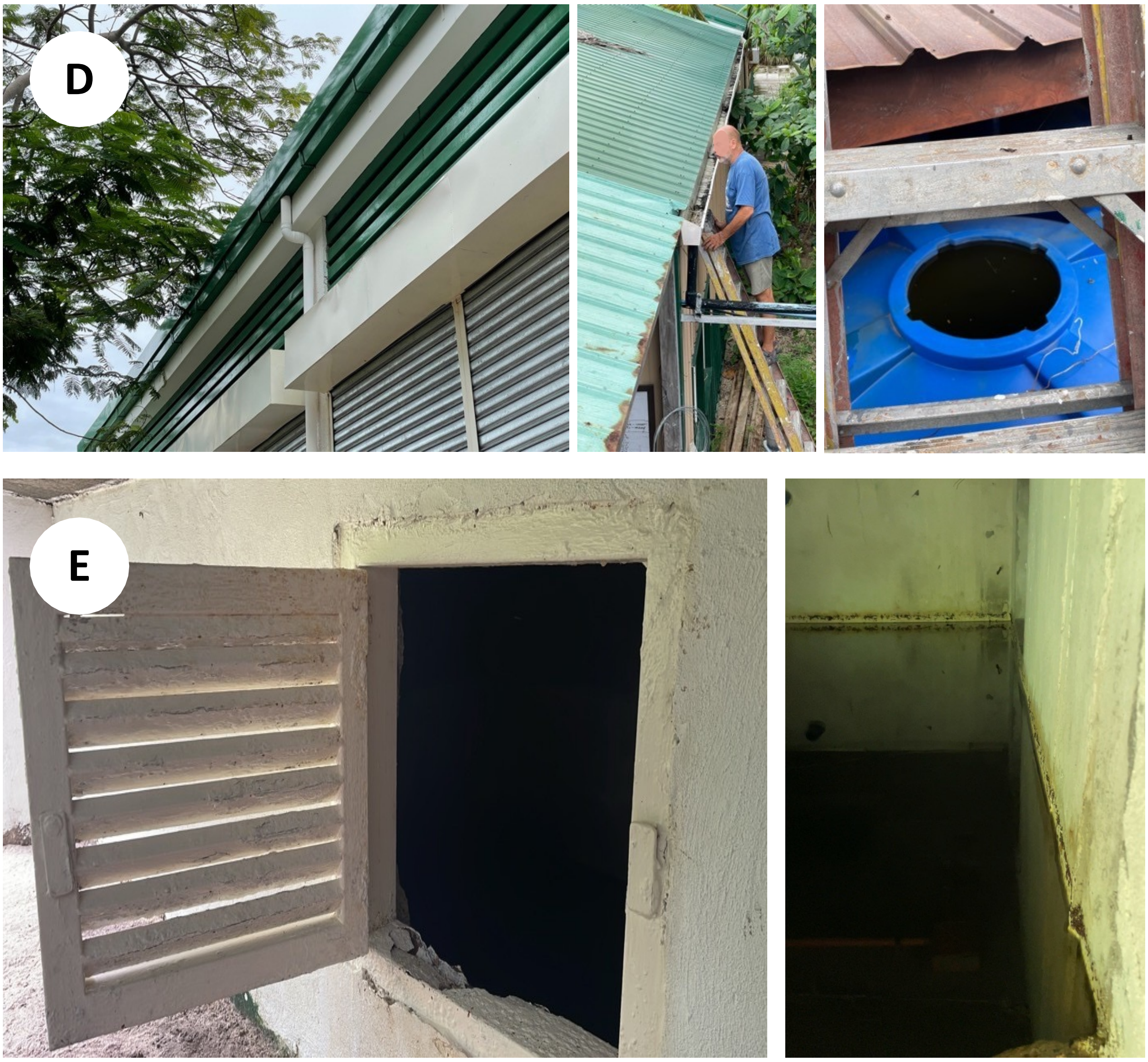

Figure S1. Mosquito breeding sites and management practices. A: Elephant ear plants (*Colocasia esculenta*) hold rain water in their leaf axils. These leaf axils were filled with soil/sand to prevent breeding; B: Used car tires are well-known mosquito breeding sites. These were either removed or filled with sand to prevent standing water; C: Miscellaneous places holding rain water such as plant pots, tarpaulins, buckets, furniture covers, or roofs. Removal or regular cleaning was practiced; D: Roof gutters that collect organic material (leaves, branches) may obstruct drainage and develop into breeding sites. Weekly inspections and cleaning was practiced; E: Underground rain water collection tanks (for sprinkling of plants) were found to harbour mosquito larvae. Proper sealing of the louvres with metal mosquito mesh prevented entry of gravid mosquitoes and exodus of newly emerged adults and solved this problem.
